# Supplementary figures and images for: The geometry of photopolymerized topography influences neurite pathfinding by directing growth cone morphology and migration
Source: bioRxiv. 2023 Aug 29:2023.08.28.555111. Preprint. [Version 1] doi: 10.1101/2023.08.28.555111 (PMC10491164; doi:10.1101/2023.08.28.555111)

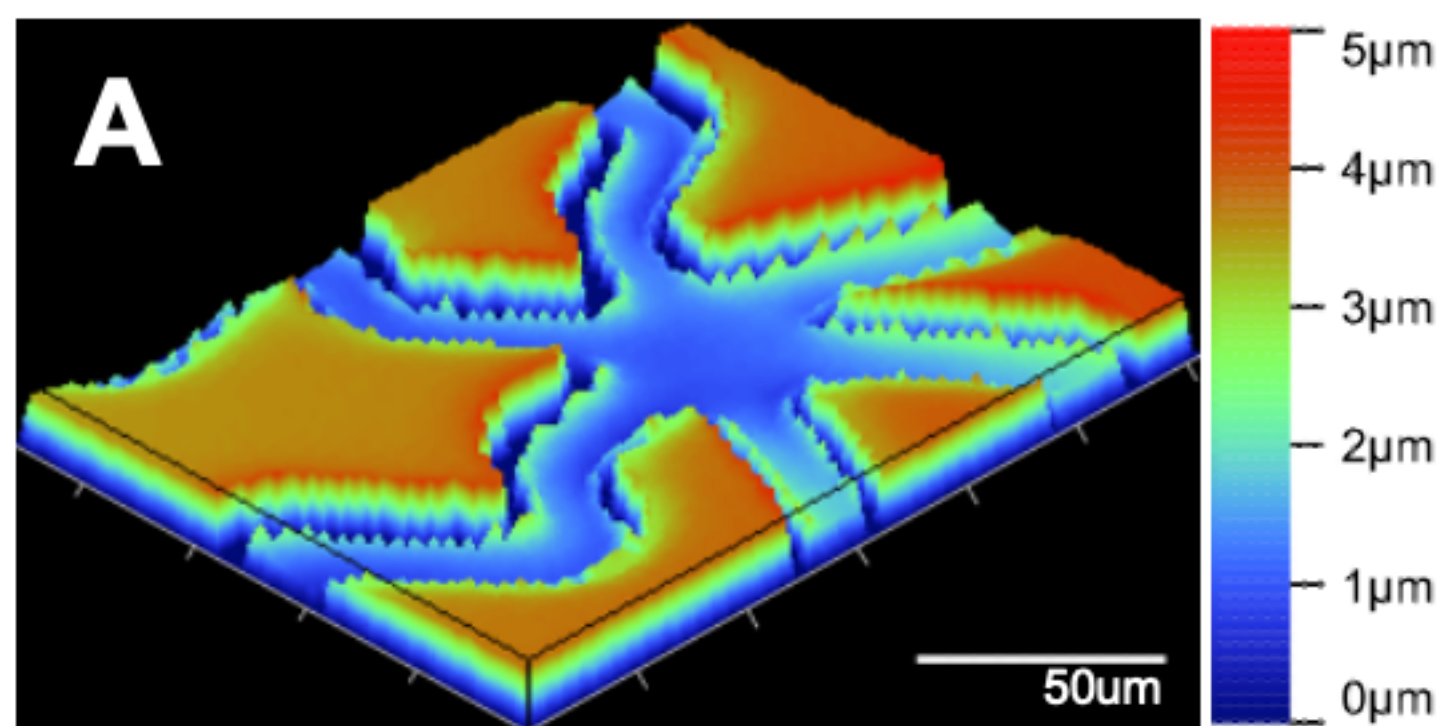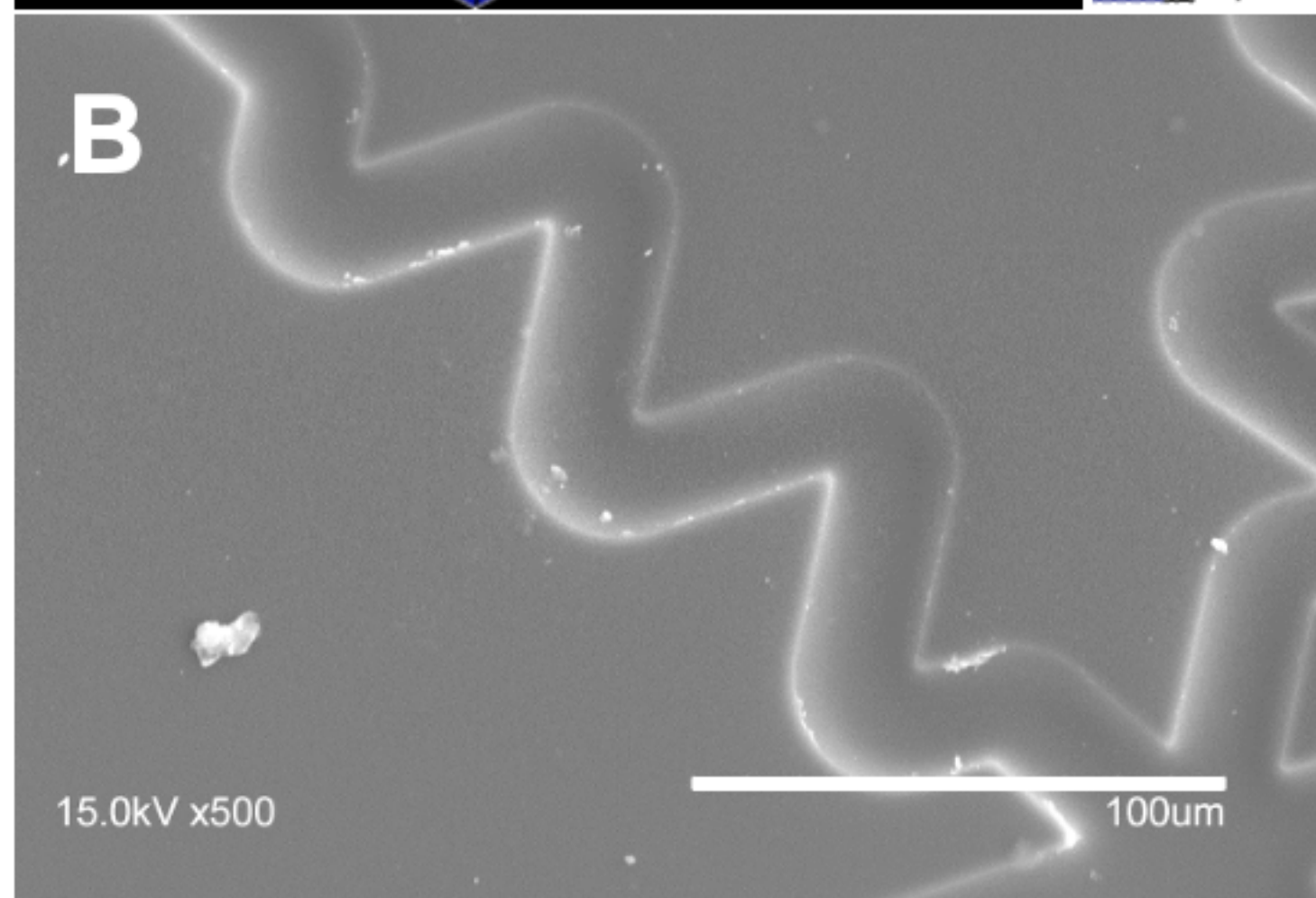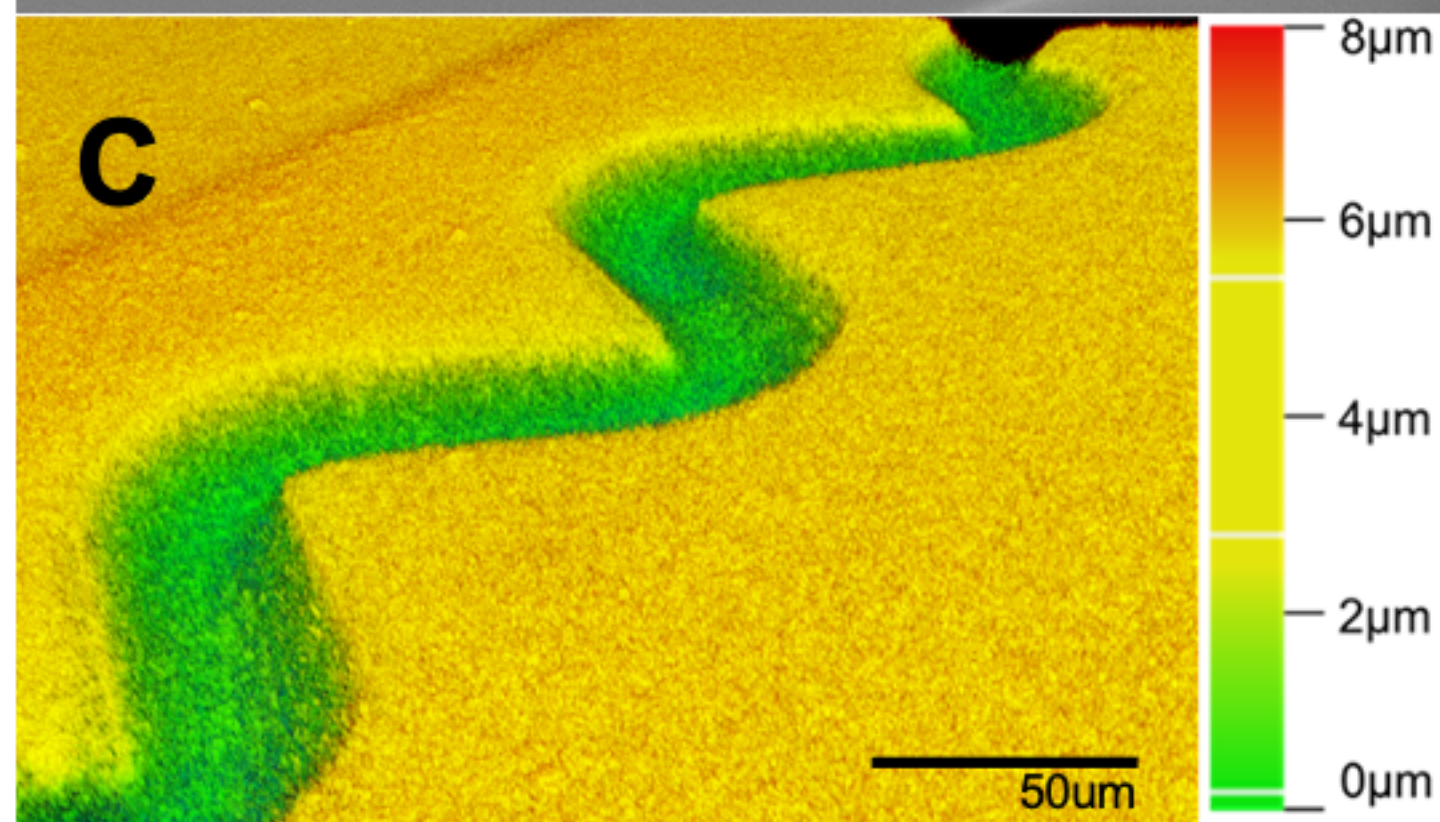

Supplement: Supplement 1 — Supplemental Figure 1: Three Methods Used to Characterize the Engineered Multi-Angled Microfeatures on the Substrate Surface. A. Depth coded white light interferometry image taken using an optical profiling system. B. Depth coded confocal microscopy image of 60° angle microfeature. C. SEM image of 60° angle microfeature and substrate surface. Scale bars = 50 μm. [file media-1.pdf]

**A**

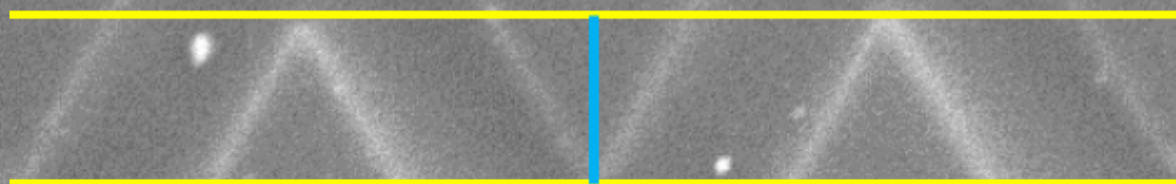

**B**

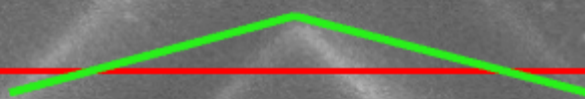

**C**

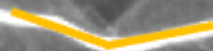

Supplement: Supplement 2 — Supplemental Figure 2: Dimensions of Interest in the Multi-Angled Microfeature Substrate. A. SEM image of 60° angle microfeature showing the 20 μm (blue) that a growth cone needs to deviate to navigate that turn. B. SEM image of 90° angle microfeature showing the measurement of the minimum angle required for a neurite to hold tension. This minimum angle was defined as the path with the most gradual angle a neurite could take from halfway down each straight segment and remain in the microfeature around a turn (151° for the 90° microfeature shown here). C. NF200 labeled SGN making and holding a turn in a 90° microfeature. Labeled in orange is the measurement of the angle the neurite makes around the turn found by the angle made with 30 μm of neurite length on each side of the turn. Scale bar = 50 μm. [file media-2.pdf]

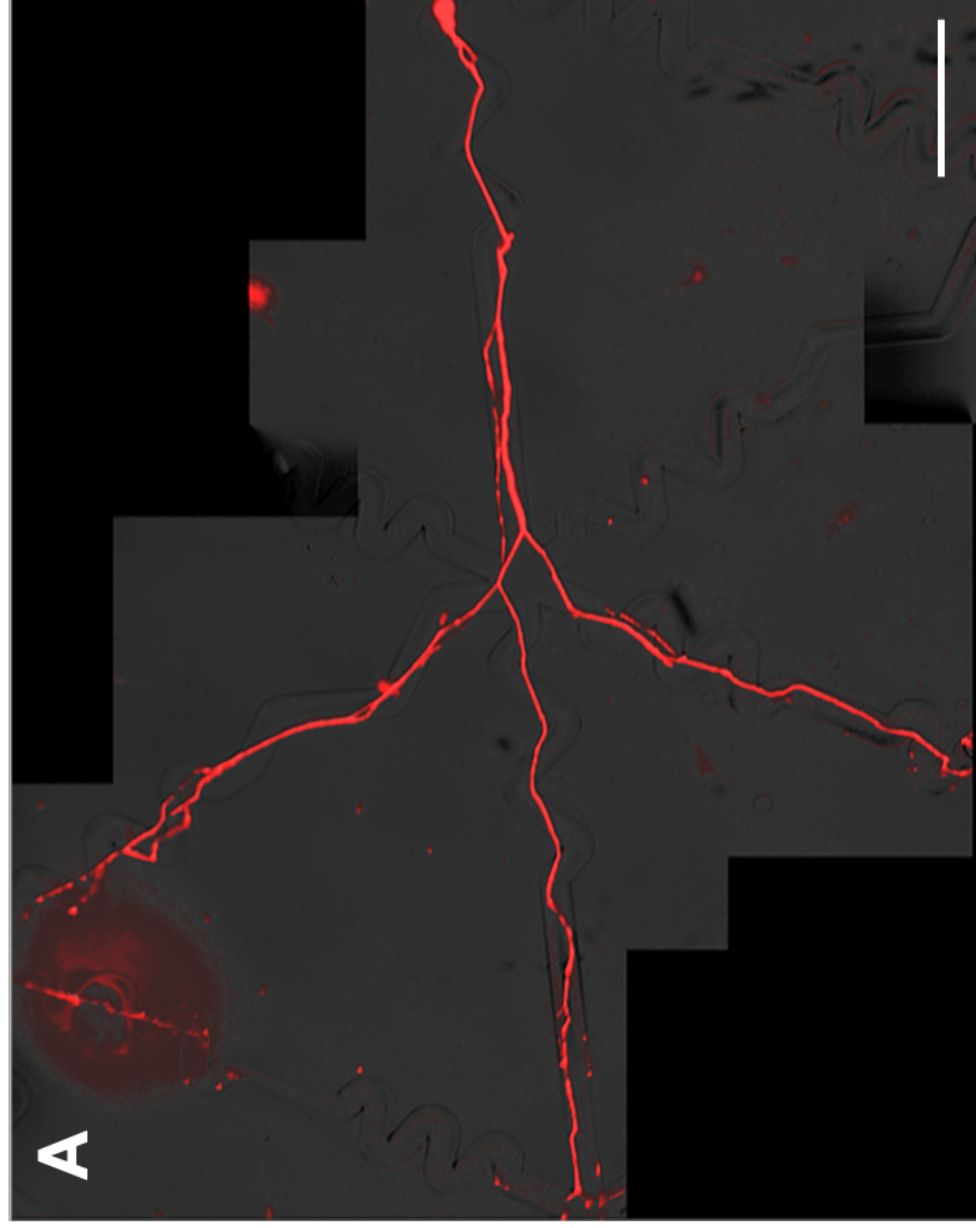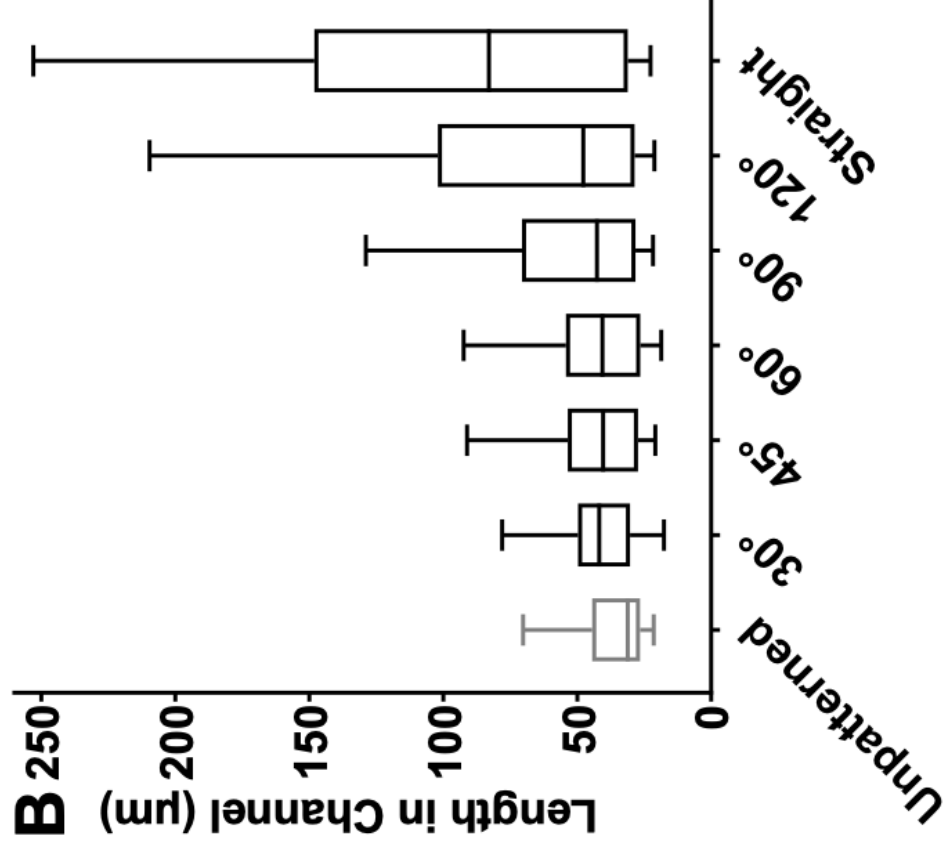

Supplement: Supplement 3 — Supplemental Figure 3: Replated DRGN Neurites Also Follow These Microfeatures in a Geometry Dependent Manner. A. Representative image of rDRGN fixed on an 8 μm substrate. B. 95% confidence interval of distance rDRGN neurite follows a microfeature once encountering it. n for each sub-condition ranges from 60 to 128. Unpatterned represents the shape of an angled microfeature overlayed onto a flat substrate. One-way ANOVA shows that distance the neurite remains in a microfeature increases with more gradual turns. p < 0.001. Scale bar = 100 μm [file media-3.pdf]

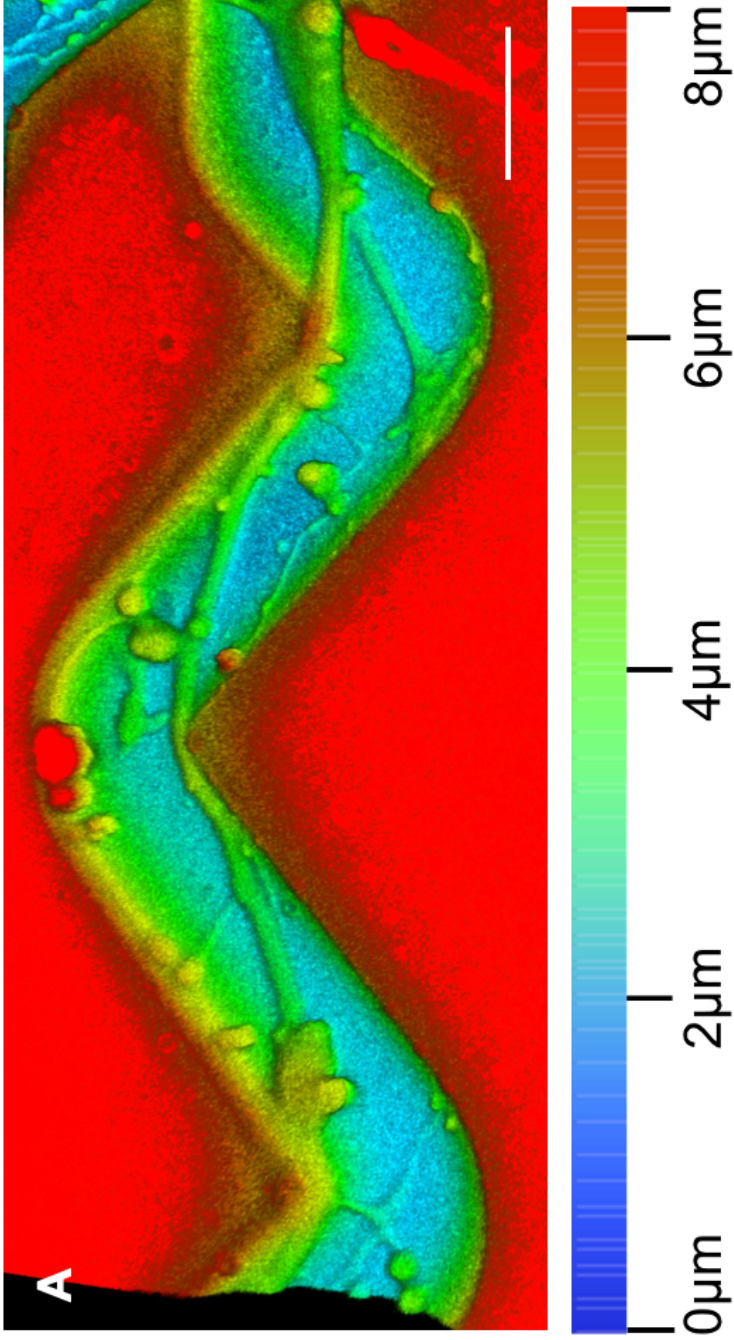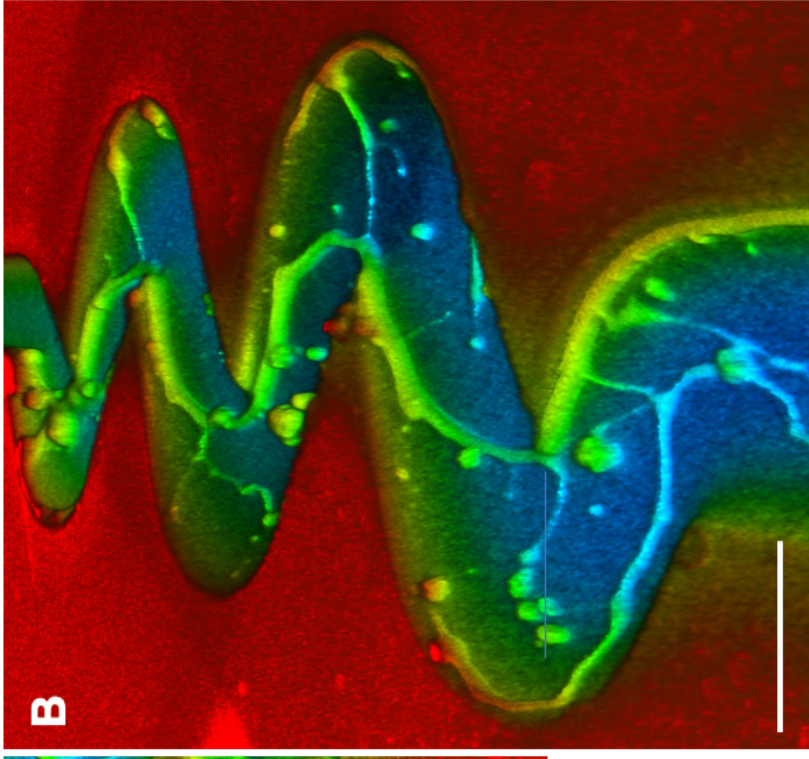

Supplement: Supplement 4 — Supplemental Table 1: Description of behavior of all growth cones imaged and assessed for Figure 4. Supplemental Figure 4: Depth Coded Confocal Images Show z-Position of Neurite Shafts in Turns. Images represent depth coded signal of combination of fluorescent signal from NF200 (568nm wavelength) and HMA/HDDMA micropatterned substrate (405nm wavelength). A. Replated DRGN neurite which partially exits the microfeature during a turn in an 8 μm amplitude, 90° angle microfeature. B. Replated DRGN neurite holding position on the sloped edge of a microfeature ridge in an 8 μm amplitude, 60° angle microfeature. Scale bar = 20 μm [file media-4.pdf]

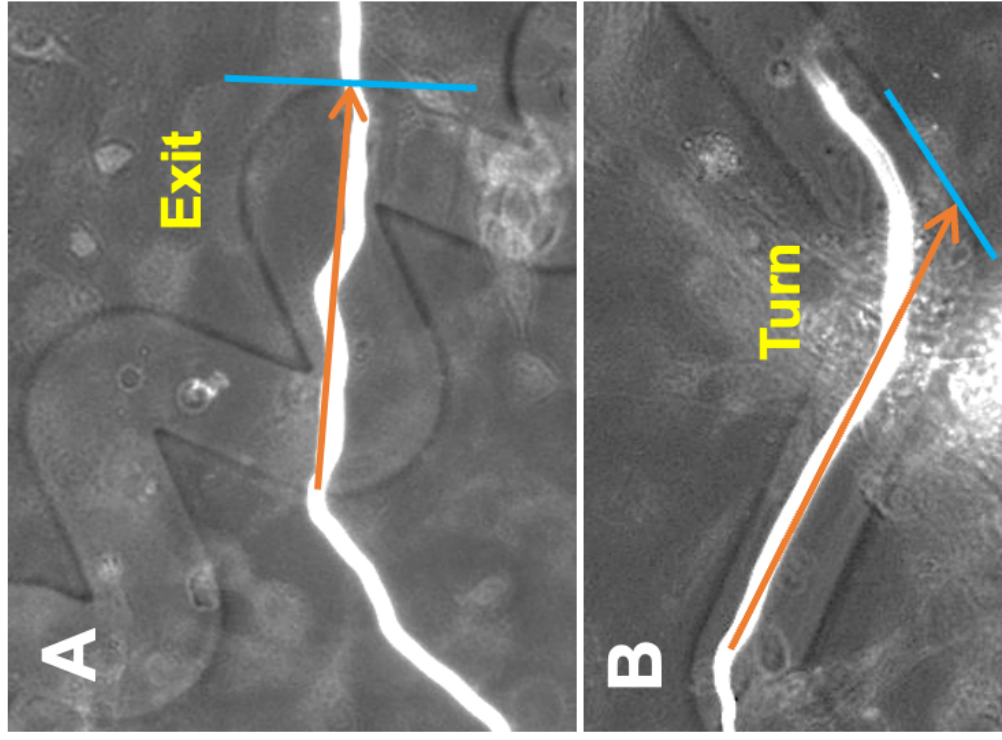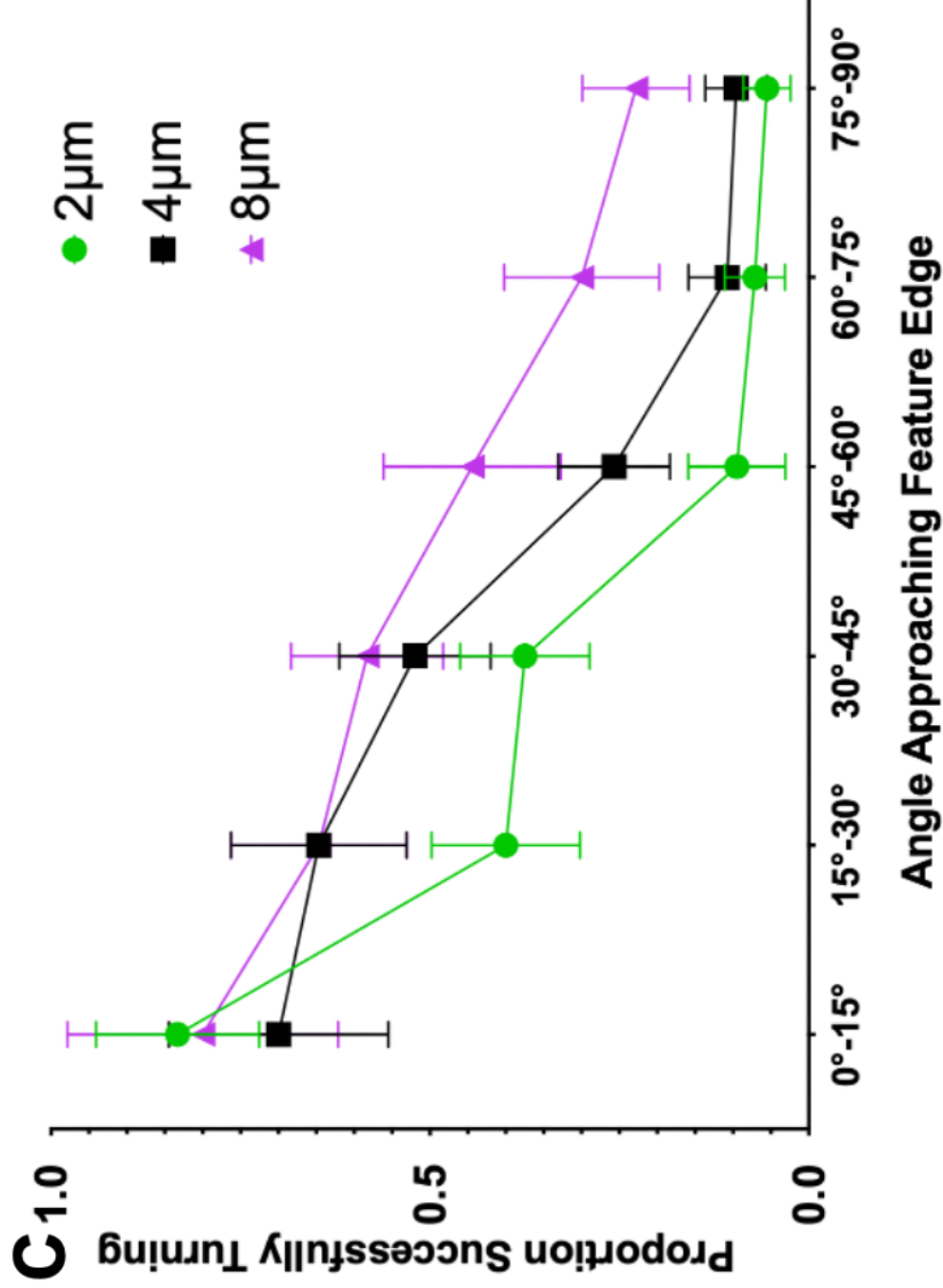

Supplement: Supplement 5 — Supplemental Figure 5: Neurite Turning is Dependent on Angle the Neurite Approaches Microfeature Wall and Microfeature Amplitude. A. Representative image of a SGN neurite approaching the edge at ~85° and exiting the microfeature. Angle is calculated from the average trajectory of the neurite approaching the edge (orange arrow) and the line tangent the edge (blue). B. Representative image of a SGN neurite approaching the at ~65° and turning. C. SGN neurite turning assessed as a function of the angle at which the neurite encounters microfeature wall, regardless of the angle of the microfeature in which the neurite was pathfinding. Two-way ANOVA shows that proportion of neurites increases with smaller angle needed to navigate the turn and with greater feature amplitude. p < 0.001 [file media-5.pdf]
